# Stearoyl-CoA Desaturase 1 as a Therapeutic Target for the Treatment of Cancer

**DOI:** 10.3390/cancers11070948

**Published:** 2019-07-05

**Authors:** Zuzanna Tracz-Gaszewska, Pawel Dobrzyn

**Affiliations:** Laboratory of Molecular Medical Biochemistry, Nencki Institute of Experimental Biology Polish Academy of Sciences, 02-093 Warsaw, Poland

**Keywords:** lipid metabolism, stearoyl-CoA desaturase 1 (SCD1), monounsaturated fatty acids, SCD1 inhibitors, targeted therapy

## Abstract

A distinctive feature of cancer cells of various origins involves alterations of the composition of lipids, with significant enrichment in monounsaturated fatty acids. These molecules, in addition to being structural components of newly formed cell membranes of intensely proliferating cancer cells, support tumorigenic signaling. An increase in the expression of stearoyl-CoA desaturase 1 (SCD1), the enzyme that converts saturated fatty acids to ∆9-monounsaturated fatty acids, has been observed in a wide range of cancer cells, and this increase is correlated with cancer aggressiveness and poor outcomes for patients. Studies have demonstrated the involvement of SCD1 in the promotion of cancer cell proliferation, migration, metastasis, and tumor growth. Many studies have reported a role for this lipogenic factor in maintaining the characteristics of cancer stem cells (i.e., the population of cells that contributes to cancer progression and resistance to chemotherapy). Importantly, both the products of SCD1 activity and its direct impact on tumorigenic pathways have been demonstrated. Based on these findings, SCD1 appears to be a significant player in the development of malignant disease and may be a promising target for anticancer therapy. Numerous chemical compounds that exert inhibitory effects on SCD1 have been developed and preclinically tested. The present review summarizes our current knowledge of the ways in which SCD1 contributes to the progression of cancer and discusses opportunities and challenges of using SCD1 inhibitors for the treatment of cancer.

## 1. Introduction

One characteristic feature of oncogenic transformation is the deep reprogramming of cellular metabolism. Discovered nearly a century ago, alterations that are defined as the Warburg effect include a higher glucose uptake and its conversion to lactate, independent of oxygen availability and mitochondria [1,2]. Numerous studies have reported an increase in fatty acid (FA) biosynthesis that results from the greater demand for these macromolecules in intensively growing and proliferating cancer cells. Research has shown alterations of lipid uptake and metabolism during tumorigenesis, which have been linked to the maintenance of cancer cell survival and metastatic potential [3]. Unlike in normal tissues, which gain energy and structural components mainly from circulating lipids, the vast majority of cancer cell lipids are synthesized de novo [4]. This phenomenon is considered to result from restrictions that are imposed by the tumor microenvironment. This mainly includes limited supplies of nutrients and oxygen that result from a poorly developed vascular system that falls behind intensive tumor growth [5]. To overcome these obstacles, cancer cells develop effective lipid metabolism machinery, including an increase in the activity of key lipogenic enzymes, such as adenosine triphosphate (ATP)-citrate lyase (ACLY), acetyl-CoA carboxylase (ACC), fatty acid synthase (FAS), and stearoyl-CoA desaturase (SCD) [6]. The high lipogenic activity of these enzymes is associated with a more advanced stage of malignancy, [7] and is driven to a large extent by the constitutive hyperactivation of oncogenic pathways. Increased de novo FA synthesis, one of the features that differentiate cancer cells from normal cells, makes them independent of limited environmental conditions, covers the demand for structural components, and represents a survival strategy to overcome hypoxic stress by providing oxidative power and improving the redox balance [8].

SCD1 is the most abundant human isoform of SCD. SCD1 is a critical player in the de novo synthesis of FAs, catalyzing the conversion of saturated fatty acids (SFAs) into ∆9-monounsaturated fatty acids (MUFAs). SFAs and MUFAs are the major components of mammalian cell lipids, including phospholipids (PLs), diacylglycerols (DAGs), triacylglycerols (TAGs), and cholesteryl esters (CEs) (i.e., basic components of biological membranes and sources of energy and signaling molecules). However, studies of the FA profile in serum and erythrocytes indicated a lower ratio between SFA and MUFA synthesis in patients with pancreatic, liver, colorectal, breast, and prostate cancer. This alteration of the SFA/MUFA ratio has been suggested to be a predictive marker in breast and prostate cancer patients [9,10,11,12,13]. Higher levels of MUFAs have also been found in transformed cells and cancerous tissue [14,15,16,17,18], and have been shown to modulate tumorigenic pathways [19,20,21]. Consistent with these findings, SCD1 expression is significantly elevated in various human cancer cells [22,23,24,25]. Moreover, this increase in SCD1 expression is positively correlated with cancer aggressiveness and poor patient prognosis in liver, thyroid, prostate, pancreatic, kidney, skin, and breast cancer [16,26,27,28,29]. SCD1 has emerged as a novel key player in tumorigenesis and may be a potential target for anticancer therapy. The present review summarizes our current knowledge of the role of SCD1 in the regulation of cancer cell metabolism and discusses the therapeutic potential of SCD1 inhibitors and challenges to their clinical use for the treatment of human cancer.

## 2. SCD1 Activity and Expression Regulation

Human SCD, also known as ∆9-fatty acyl-CoA desaturase, is an endoplasmic reticulum-associated enzyme that catalyzes the introduction of a double bond in the cis-∆9 position of saturated fatty acyl-CoAs. The primary substrates of SCD are palmitoyl-CoA and stearoyl-CoA, which produce palmitoleoyl-CoA and oleoyl-CoA, respectively [30,31]. Two isoforms of SCD are found in human tissues: SCD1 and SCD5. SCD1 is the main isoform that is widespread in all types of cells, with the highest levels in adipose tissue, brain, heart, liver, and lungs. The expression of SCD5 is limited in adult human tissues and appears to be mostly restricted to the brain and pancreas [26,32,33,34]. Human SCD1 expression is mainly modulated by transcriptional regulation, supported by the observation that the promoter of the *SCD* gene contains many consensus binding sites for transcription factors that are involved in the regulation of lipogenic pathways [35]. However, protein degradation pathways are also implicated in the modulation of SCD1 activity [36,37,38]. Two main pathways that activate lipogenesis can be distinguished: The insulin and glucose signaling pathways. Sterol regulatory element binding protein 1 (SREBP1) and carbohydrate response element binding protein (ChREBP) are the main drivers of these pathways, respectively. Three isoforms of SREBP are expressed in human tissues: SREBP1a, SREBP1c, and SREBP2, encoded by two separate genes [39]. The SREBP1c isoform mainly drives FA synthesis, whereas the function of SREBP2 is limited to the regulation of genes that are involved in cholesterol biosynthesis and embryonic development. The SREBP1a isoform is implicated in both of these lipogenic pathways [40,41,42]. SREBP1 deficiency results in a lower content of unsaturated lipids and causes the apoptotic death of cells with limited access to exogenous lipids [43]. Unlike SREBP1, the activation of ChREBP is induced by intermediates of glucose metabolism via multiple insulin-independent mechanisms [44,45,46]. SREBP1 and ChREBP clearly act synergistically in the induction of SCD1 and the expression of other lipogenic genes in response to glucose and insulin, respectively [47,48]. However, tight regulation of the desaturation reaction is a more complex process, reflected by various transcription factors that bind to the *SCD* promoter, notably peroxisome proliferator activated receptor α (PPARα), liver X receptor (LXR), CCAAT/enhancer binding protein α (C/EBP-α), nuclear transcription factor Y (NF-Y), neurofibromin 1 (NF-1), and specificity protein 1 (SP1), all of which are activated by various growth factors, cytokines, hormones, and nutritional status [49]. Leptin is an adipocyte hormone that regulates energy homeostasis [50] and suppresses SCD1 expression by enhancing the binding of SP1 and activator protein 1 (AP-1) transcription factors to leptin response element (LepRE) that is located in the *SCD1* promoter, surpassing the stimulation by insulin [51]. The inhibitory effect of leptin on SCD1 may also result from the negative regulation of SREBP-1c through the leptin-driven activation of signal transducer and activator of transcription 3 (STAT3) [52,53,54]. Estrogen, glucagon, and thyroid hormone T3 were shown to negatively impact SCD1 expression. The inhibitory effect of nutritional status on SCD1 is mainly driven by polyunsaturated fatty acids (PUFAs) through the modulation of SREBP-1c, NF-Y, PPARs, and LXR that bind to the *SCD1* promoter. PUFAs were also shown to suppress SCD1 expression via the extracellular regulated kinase/mitogen activated protein kinase (ERK/MAPK) signaling pathway [35].

## 3. SCD1 and Lipid Metabolism in Cancer Cells

Dividing cells must double their reservoir of FAs to maintain their proper content in daughter cells. Fatty acids are macromolecules that are primarily used as structural components, energy stores, and signaling lipids. Intensively proliferating cancer cells are distinguished by the greater demand for MUFAs, which are utilized mainly for the synthesis of new membrane-forming PL, TAG, and CE [55]. An increase in the content of lipids that are enriched with MUFAs (mostly phosphatidylcholine) and the simultaneous reduction of the levels of SFAs and PUFAs have been found in tumor tissues of different origins (e.g., breast, lung, colorectal, gastric, esophageal, and thyroid cancer) [18]. The observed accumulation of MUFAs overlaps with higher levels of SCD1 in cancerous tissue [18,56]. A detailed metabolic analysis of pancreatic ductal adenocarcinoma (PDAC) tumors revealed higher levels of palmitoleate and oleate in cells of an aggressive subtype [57]. Analyses of tumor tissue samples that were collected from breast and hepatocellular carcinoma (HCC) patients showed an association between high SCD1 expression and shorter survival [16,24]. Thus, these and other studies clearly demonstrate that the shift toward an increase in SCD1 activity is specific to various types of cancer and correlates with their aggressiveness and poor patient prognosis.

Further studies demonstrated that the stable knockdown of SCD1 in SV40-transformed human lung SV40-WI38 fibroblasts decreased MUFA and phospholipid synthesis, decreased the rate of cell proliferation, and induced apoptosis [58]. Similarly, the inhibition of SCD1 activity led to cancer cell death through the depletion of MUFAs [59,60]. In addition to driving effects that strictly depend on MUFA synthesis, SCD1 also modulates other lipid metabolism pathways that are implicated in tumorigenesis. A recent analysis of data from The Cancer Genome Atlas (TCGA) showed that 806 genes were associated with SCD1 overexpression in human colon cancer samples. These genes are required for lipid synthesis and incorporation, among other functions. Furthermore, an in silico ingenuity pathways analysis revealed the SCD1-dependent regulation of FA, TAG, cholesterol, and PL synthesis through the stimulation of SREBPs [61]. These observations provided strong evidence that SCD1 and products of its catalytic activity are crucial drivers of neoplastic transformation and cancer progression. Therefore, the application of SCD1 inhibitors as possible anticancer agents may be justified. The design of more potent SCD1-related compounds may be facilitated because the structure of SCD1 has been resolved [62,63].

## 4. SCD1, Cancer Cell Proliferation, and Tumor Growth

SCD1 was shown to be a potential anticancer therapeutic target. At that time, however, no selective compounds were known to provide a starting point for the development of SCD1 inhibitors. The high-throughput screening of large chemical libraries emerged as the most rational method for this purpose. The first patents that described small-molecule SCD1 inhibitors were submitted by Xenon Pharmaceuticals in 2005. These compounds included piperazinylpyridines and nicotinamide or pyridazine derivatives as potent inhibitors for the treatment of diabetes and other SCD1-mediated diseases [64]. Since that time, many inhibitors of SCD1 emerged, based on the chemical scaffolds that are mentioned above (Table 1). In 2008, Abbott Laboratories developed A939572, an orally available piperidine-aryl urea-based small molecule [65]. A939572 appeared to be a very potent SCD1 inhibitor that has been broadly investigated in cancer research in both in vitro and in vivo models. A939572 was shown to markedly reduce the proliferation of lung and pharynx cancer cells but only in serum-reduced conditions. This effect was abrogated by the addition of exogenous monounsaturated oleic and palmitoleic acid or polyunsaturated linoleic acid, confirming that the sensitivity of intensively proliferating cancer cells to SCD1 inhibition results from the limited availability of MUFAs [60]. Emerging evidence supports this thesis, in which the desaturation pathway is tightly regulated during cell cycle progression. Many hormones and growth factors, such as transforming growth factor β (TGF-β*)*, epidermal growth factor receptor (EGFR), fibroblast growth factor receptor 3 (FGFR3), and platelet-derived growth factor (PDGF), induce the expression of SCD1 [66,67,68,69,70]. Further studies showed that the A939572-induced inhibition of SCD1 suppresses the proliferation and induces the apoptosis of cancer cells of different origins, including the kidneys, bladder, liver, colon, thyroid, and endometrium [27,71,72,73,74,75]. Similar effects were reported for other inhibitors of SCD1, including CAY10566, MF-438, and CVT-11127 in the case of breast, lung, and colorectal cancer cells [74,76,77,78]. The inhibition of SCD1 by CVT-11127 inactivated the EGFR-dependent mitogenic pathway and impaired the EGF-mediated proliferation of H460 lung cancer cells. This study also showed that desaturase inhibition decreased the mobility of fluid lipid domains in the plasma membrane of H460 cells, demonstrating that MUFA synthesis is essential for the undisturbed proliferation of cancer cells [79]. The SCD1 inhibitor, CVT-11127, also arrested H460 cells in the G1/S stage of the cell cycle and triggered programmed cell death. Importantly, the blockade of SCD1 activity with CVT-11127 did not impair the proliferation of normal human fibroblasts, which confirms a lower demand for endogenously synthesized MUFA in non-cancer cells [76].

SCD1 inhibition by chemical compounds has also been shown to suppress the growth and proliferation of cancer cells in animal models. Kim et al. reported that SCD1 overexpression stimulated human prostate tumor growth in a mouse xenograft model [80]. The SCD1 inhibitor A939572 significantly reduced the volume of primary human gastric cancer and colorectal cancer xenografts in mice, with no effect on body weight [60,74]. Additionally, Fritz et al. reported that administration of the SCD1 inhibitor, BZ36, a nicotinamide derivative, in mice that bore prostate cancer xenografts slowed tumor growth and generated a limited population of tumor cells that were positive for the proliferation-associated marker proliferating cell nuclear antigen (PCNA) [81]. A novel orally available piperidine derivative, T-3764518, also dose-dependently inhibited the growth of human colorectal cancer and mesothelioma cancer xenografts in mice. An analysis of tumor lipidomic profiles revealed 54 lipid molecules (with a prevalence of phosphatidylcholine and diacylglycerols), with a lower desaturation index in animals that were treated with T-3764518 [82]. This small molecule with a promising pharmacokinetic profile also reduced tumor volume in a mouse xenograft model of human renal cell adenocarcinoma [83].

## 5. SCD1, Hypoxia, Endoplasmic Reticulum Stress, and Cancer Cell Apoptosis

Tumor cells are anchored in an oxygen and nutrient-restricted environment. The de novo desaturation of FAs by desaturases requires unlimited access to oxygen as the terminal acceptor of electrons. A restricted supply of oxygen under conditions of tumor-like ischemic stress inhibits the reaction of desaturation and renders mouse embryonic fibroblast cells dependent on exogenous unsaturated FAs or leads to programmed cell death when access to these macromolecules is limited [96]. The death of hypoxic cells that are exposed to unsaturated lipid deficiency results from the activation of endoplasmic reticulum (ER) stress that is mediated by the unfolded protein response (UPR), a process that is initiated by the ER-associated transmembrane sensors inositol-requiring enzyme 1 (IRE-1), protein kinase R (PKR)-like endoplasmic reticulum kinase (PERK), and activating transcription factor 6 (ATF6) [97]. The apoptotic response that is induced by ER stress results from integrated pathways that are downstream of IRE-1 and CCAAT-enhancer-binding protein homologous protein (CHOP*)*, involving crucial effectors of cell death, such as BCL2-associated X protein/BCL-2 homologous antagonist/killer (BAX/BAK), c-Jun N-terminal kinase (JNK), and Ca^2+^/calmodulin-dependent protein kinase II (CaMKII) [98]. The ER stress response is activated by ER membrane disruption that is caused by an increase in the incorporation of saturated phospholipids and can be overcome by supplementation with unsaturated FAs [99,100]. Thus, the inhibition of SCD1 activity induces ER stress and consequently apoptosis in multiple cancer cell lines, with no effect on normal cells. The treatment of renal, thyroid, liver, and lung cancer cells with the SCD1 inhibitor, A939572, mostly under restricted nutrient conditions, upregulates a set of gene markers of UPR and ER stress, including CHOP, binding immunoglobulin protein (BiP), Tribbles pseudokinase 3 (TRIB3), ATF6, DNA damage-inducible transcript 3/4 protein (DDIT3/4), homocysteine inducible ER protein with ubiquitin like domain 1 (HERPUD1), and growth arrest and DNA damage-inducible α (GADD45α) [27,60,71,73]. Similar observations have been made after the inhibition of SCD1 with T-3764518 in colorectal cancer cells and upon the administration of MF-438 and CVT-11127 in bone osteosarcoma cells [82,101]. The SCD1 inhibitor, A939572, also induced the ER stress response in vivo in a xenograft mouse model of human clear cell renal cell carcinoma (ccRCC) [71]. These data strongly support the notion of anticancer strategies that are based on pharmacological targeting of SCD1 because of the extreme sensitivity of cancer cells to unsaturated lipid deprivation that triggers cell death via ER stress and the UPR. Moreover, Zhang et al. found that the expression of SCD1 under conditions of hypoxic stress was regulated by hypoxia-inducible factor 2α (HIF-2α), revealing oncogenic properties [102]. A positive feedback loop was found between HIF-2α and SCD1 that was mediated by the phosphoinositide 3-kinase/protein kinase B (PI3K/AKT) pathway. Both proteins were shown to mutually enhance their function, resulting in synergistic effects on modulation of the growth, survival, and migration of ccRCC cells. These authors claimed that pharmacologically targeting both SCD1 and HIF-2α may be an effective approach to treat this type of cancer [102]. Interestingly, Chen et al. demonstrated that apoptotic death of colorectal cancer cells treated with A939572 SCD1 inhibitor is mediated by intracellular ceramide signals powered by increased ceramide biosynthesis, rather than SFA lipotoxicity [74]. Induction of ER-stress mediated apoptosis by disruption of ER calcium homeostasis in cancer cells is one of the apoptotic pathways stimulated by ceramides [103,104].

## 6. SCD1 and Cancer Cell Migration, Invasion, and Metastasis

Cancer cells that form a solid tumor mass present an epithelial phenotype, distinguished by defined cell polarity and strong cell–cell adhesion, driven mainly by E-cadherin. This protein has been recognized as a suppressor of cell invasion, in which the downregulation of E-cadherin was sufficient to direct most cells toward the mesenchymal differentiation program [105,106]. This process occurs in several stages of embryogenesis and during tissue regeneration, organ fibrosis, and wound healing [107]. However, the epithelial–mesenchymal transition (EMT) has implications for the pathogenesis of cancer cells with regard to the acquisition of invasive and metastatic potential that is correlated with a decrease in patient survival [108,109]. The EMT is a process that can be distinguished by radical changes in molecular profiles and cell morphology. This process is also accompanied by significant changes in lipid metabolism. Higher levels of FA uptake were observed in hepatocellular carcinoma cells that underwent the EMT [110]. The induction of this process by tumor necrosis factor a (TNF-α), a proinflammatory cytokine that is associated with an increase in metastasis in human prostate cancer, was shown to lead to the accumulation of unsaturated TAGs in the DU145 prostate cancer cell line [111]. The inhibition of de novo FA synthesis in *Src*-transformed NIH/3T3 mouse embryonic fibroblasts impaired the formation of invadopodia, membrane structures that are involved in cell migration. This effect was abolished by supplementation with oleic acid, the main product of SCD1 activity [112]. Finally, elevations of the levels of SCD1 have been shown to promote the migration and invasion of cancer cells by increasing the level of MUFAs [21]. Blockade of the activity of SCD1 with A939572 suppressed the migration and invasion of HCC cells [73] and poorly or highly invasive breast cancer cells (MCF-7 and MDA-MB-231, respectively). Oleic acid restored the original migration ability of A939572-treated MCF-7 and MDA-MB-231 cells [84]. One mechanism of the SCD1-driven EMT is the dysregulation of β-catenin function. β-catenin is a component of adherent junctions and interacts with E-cadherin that is anchored to the cell membrane [113]. Cytosolic fractions of β-catenin are rapidly degraded by the proteasome system in a glycogen synthase kinase 3β (GSK3β)-dependent manner. The loss of E-cadherin leads to the inhibiting phosphorylation of GSK3β, and β-catenin is thus not degraded and translocates to the nucleus, where it activates a set of genes, the expression of which promotes cell motility and invasiveness. SCD1 deficiency suppresses GSK3β phosphorylation, and, in turn, β-catenin is degraded. Consequently, the cell acquires a more epithelial phenotype. Thus, SCD1 was suggested to be a therapeutic target for the treatment of metastatic breast cancer [114]. Additionally, Scalia and Igal demonstrate that SCD1 knockdown impairs GSK3β phosphorylation via the inhibition of AKT phosphorylation in A549 lung cancer cells. Supplementation with exogenous oleic acid restored AKT phosphorylation in mock-treated cells, but not in cells with SCD1 deficiency, which suggests that fatty acid-mediated stimulation of AKT signaling in A549 cells requires active MUFA synthesis [115]. Another study implicated SCD1 in the higher mortality of colorectal cancer (CRC) patients with comorbid diabetes. Meta-analyses and pathologic analyses showed increases in tumor invasion, staging, and mortality of CRC patients with diabetes compared with CRC patients without diabetes [116,117]. SCD1 upregulation was also shown to be a risk factor for CRC and that the migration and invasion of CRC cells are stimulated by MUFAs via inhibition of the phosphatase and tensin homolog (PTEN)/AKT pathway [21,118]. SCD1 is a crucial factor in the pathogenesis of type 2 diabetes, in which it is a downstream effector of glucose sensing by ChREBP [119]. High glucose levels lead to the ChREBP-mediated upregulation of SCD1, which in turn induces the EMT in HCT116 CRC cells [21].

Sanchez-Martinez et al. reported that the observed SCD1-dependent increase in colon cancer cell migration and invasiveness resulted from the synergistic action of SCD1 with the long-chain acyl-CoA synthetases acyl-CoA synthetase long-chain family member 1 (ACSL1) and ACSL4 [120]. ACSL comprises a subfamily of enzymes that convert FAs to the active form of acyl-CoA [121]. The simultaneous overexpression of these three proteins induces the EMT and increases the migration, invasion, and survival of CRC cells to a greater extent than proteins that are overexpressed individually. Clinical data corroborate these findings, in which stage-II colorectal cancer patients who presented the overexpression of all three of these proteins in tumor samples had worse clinical outcomes compared with patients with elevated levels of only ACSL1, ACSL4, or SCD1 alone. In vitro studies showed that the combined treatment of different CRC cell lines with the ACSL and SCD1 inhibitors, Triacsin C and A939572, respectively, decreased cell viability compared with the application of either of these inhibitors alone. This inhibitory effect was particularly pronounced in the case of SW620–5FU-R colorectal cancer cells, which are resistant to conventional chemotherapy with 5-fluorouracil [120].

Angelucci et al. performed co-culture experiments and found that cancer-associated fibroblasts (CAFs), a major component of breast tumor stroma, promoted the invasiveness of poorly (MCF-7) and highly (MDA-MB-231) invasive breast cancer cells through induction of the EMT, increased membrane fluidity, and accelerated cell migration [122]. Co-culture with CAFs and, to a lesser extent, normal fibroblasts (NFs) induced SCD1 expression in the breast cancer cells that are mentioned above. The siRNA-based or pharmacological abrogation of SCD1 with A939572 impaired CAF-driven cancer cell migration, which was restored by treatment with oleic acid [84,85]. The CAF-secreted diffusible factors, hepatocyte growth factor (HGF), TGF-β, and basic fibroblast growth factor (bFGF), were shown to mediate the observed increase in cancer cell motility [85]. Cancer-associated fibroblasts induced upregulation of the SCD5 isoform in co-cultured MCF-7 cells, with no influence on E-cadherin expression or migration ability. However, SCD5 knockdown caused the necrosis of MCF-7 cells, suggesting the involvement of CAF-induced SCD5 expression in the maintenance of breast cancer cell survival [84].

A similar co-culture approach led to the conclusion that stromal components at the site of metastasis also support cancer cell colonization and survival. Murine lung fibroblasts (LFs) promoted the proliferation of murine B16F10 melanoma cancer cells in co-culture. Furthermore, the co-injection of these two types of cells in mice increased the metastasis of B16F10 cells to the lungs. Targeting SCD1 with the inhibitor, CAY10566, decreased the rate of lung metastasis and prolonged overall survival in mice. Lung fibroblast-mediated cancer cell proliferation and metastatic colonization are driven by SCD1-dependent changes in FA composition, reflected by an increase in the MUFA/SFA ratio. The same study demonstrated that LF-derived cathepsin B (CTSB) induced SCD1 overexpression in B16F10 cells via annexin A2 (ANXA2) and the PI3K/Akt/mammalian/mechanistic target of rapamycin (mTOR) pathway, which is critical for cellular metabolism and cancer progression [29]. 

The role of SCD1 in the metastatic potential of cancer cells and effectiveness of SCD1 inhibition in diminishing this aggressive phenotype appear to be indisputable. The application of SCD1 inhibitors, demonstrated by both in vitro and in vivo models, may prevent the progression of cancer to more malignant forms or restrict the colonization of its metastatic niche (Table 1).

## 7. SCD1 Inhibitors in Combined Therapy

### 7.1. SCD1 Inhibitors and Chemoresistance of Cancer Cell

A major obstacle to effective anticancer therapy is the resistance of cancer cells that is frequently acquired during the course of chemotherapy and leads to cancer relapse and dissemination [123]. Therefore, novel approaches to understanding the molecular mechanisms of cancer need to be found, and new therapeutic targets need to be discovered. SCD1 appears to be a promising molecular target of cancer chemotherapy, based on its crucial role in tumor progression and the efficient response of cancer cells to SCD1 inhibitors that are applied both in vitro and in vivo. A combined pharmacological approach that involves new molecular targets may counteract the cancer cell chemoresistance and enhance the therapeutic efficacy of commonly used chemotherapeutic drugs. For example, SCD1 overexpression was shown to be associated with the clinical resistance of melanoma cells to the B-Raf proto-oncogene, serine/threonine kinase (BRAF) inhibitor, vemurafenib [87]. Nashed et al. reported that the treatment of H460 non-small cell lung cancer (NSCLC) cells with the SCD1 inhibitor, CVT-11127 or CVT-12012, potentiated the gefitinib-dependent inhibition of cancer cell proliferation [79]. Gefitinib is an EGFR inhibitor that was approved in 2003 by the United States Food and Drug Administration for the treatment of NSCLC [124]. It was shown that SCD1 inhibition blocked EGFR autophosphorylation, which impaired the AKT/mTOR and ERK mitogenic and oncogenic pathways. The perturbation of EGFR activation by SCD1 inhibition may result from alterations of the mobility of plasma membrane lipid domains that are involved in the activation of signaling platforms on the plasma membrane. Thus, the strong inhibitory effect of SCD1 inhibitor + gefitinib combination therapy may result from the synergistic suppression of EGFR downstream targets [79]. Moreover, the combined treatment of ccCRR cells with the SCD1 inhibitor, A939572, and temsirolimus (i.e., an mTOR inhibitor for advanced renal cell carcinoma) effectively inhibited cell proliferation colony formation, and decreased xenograft tumor volume by 60%. With monotherapy, only a 20% to 30% reduction of tumor volume was observed [71]. The SCD1 inhibitor, A939572, was also combined with temozolomide (TMZ) against the TMZ-resistant glioblastoma cell lines, T98G-R and U87-R. Temozolomide is an alkylating agent that is used as the standard treatment of glioblastoma. SCD1 was shown to be significantly overexpressed in T98G-R and U87-R cells and to drive their resistance to TMZ by stimulating the Akt/GSK3β/β-catenin signaling pathway, which is relevant for cancer cell survival and the invasive phenotype. The application of A939572 sensitized cells to TMZ treatment in an in vitro model, thus demonstrating that the blockade of SCD1 overcame the chemoresistance of these cancer cells [86] (Table 1). SCD1 inhibition in cancer cells induces ER stress, activation of the UPR, and apoptosis. Endoplasmic reticulum-associated degradation (ERAD) is a pro-survival component of the UPR [27]. Thus, blocking the action of the proteasome leads to the accumulation of misfolded proteins in the ER and the induction of apoptosis. The application of proteasome inhibitors has been proposed to be a pharmacological treatment for anaplastic thyroid carcinoma (ATC), an aggressive and drug-resistant type of cancer [125,126]. Von Roemeling et al. suggested that combined treatment with SCD1 inhibitors and proteasome inhibitors may enhance the ER stress response, thus leading to the death of ATC cells. These authors found that simultaneous administration of the SCD1 inhibitor, A939572 or MF-438, with the proteasome inhibitor, bortezomib or carfilzomib, impaired the proliferation of THJ29T and THJ16T ATC cells and induced apoptosis. Generated combination index (CI) values confirmed a synergistic mode of action of the applied compounds. These results correspond with in vivo observations, in which a combination of MF-438 with carfilzomib inhibited tumor growth in a THJ16T xenograft model in mice. No effect of MF-438 alone and only a weak effect of carfilzomib monotherapy was found on tumor growth [27].

### 7.2. Cancer Cell Resistance to SCD1 Inhibitors

A distinct mechanism has been shown to render cancer cells partially resistant to the suppression of SCD1 activity. The treatment of cancer cells with the SCD1 inhibitor, CVT-11127, caused adenosine monophosphate-activated protein kinase (AMPK) activation via phosphorylation at Thr172, which in turn led to inactivation of the phosphorylation of ACC at Ser79 [78,91]. The carboxylation of acetyl-CoA by ACC generates malonyl-CoA, the substrate for chain elongation in FA biosynthesis [127]. The suppression of FA synthesis in response to SCD1 inhibition has been postulated to protect cells from the excessive accumulation of SFAs, whereas the synthesis of MUFAs is impaired [78,91]. The mechanisms of the lipotoxicity of SFAs and cytoprotective effect of MUFAs were thoroughly discussed by Nolan and Larter [128]. The inhibition of AMPK activity with compound C further reduced the proliferation of H1299 and A549 lung cancer cells that were treated with the SCD1 inhibitor, CVT-11127 [78]. Combination screening with a biologically annotated compound library identified antagonists and agents that act synergistically with the SCD1 inhibitor, T-3764518. The treatment of HCT-116 colorectal carcinoma cells with compound 7a (i.e., an inhibitor of ACC that was identified in the compound library screen) abolished the anti-growth activity of T-3764518 and revealed the cytoprotective properties of ACC attenuation after SCD1 inhibition. A similar effect was observed after administration of the FAS inhibitor, GSK2194069, showing that blockade of the FA synthesis cascade contributed to the resistance of cancer cells to SCD1 inhibition. Interestingly, suppressors of autophagy (i.e., STA5326, a Bax channel blocker, vacuolin-1, and hydroxychloroquine) had a synergistic mode of action with T-3764518 (i.e., potentiated its anti-growth activity) in HCT-116 cells [91]. Activated AMPK is a key positive regulator of autophagy [129,130]. The AMPK-mediated induction of this process has been suggested to provide an escape route from the cytotoxic effects of SCD1 inhibition. The perturbation of FA synthesis and induction of autophagy guarantee, to some extent, the survival of SCD1-knockout HCT-116 cells [91]. These observations led to the conclusion that anticancer therapeutic strategies that consist of SCD1 inhibition may be potentiated by combination therapy that targets AMPK-dependent pathways.

However, autophagic events, which usually provide protection against different cellular stressors, may also act as cell death effectors. This occurs, for example, during mammalian embryogenesis or in the case of cells with impairments in apoptotic pathways [131]. Huang et al. found that SCD1 inhibition with CAY10566 induced the AMPK-mediated death of HCC cells via autophagy. These authors observed the accumulation of cytoprotective autophagy-associated p62 protein in HCC cell lines or tumor samples that overexpressed SCD1. The level of p62 decreased after SCD1 inhibition [89,132]. Additionally, the tumorigenic activity of p62 and its contribution to cancer cell chemoresistance were reported [133]. Thus, the differential expression of autophagic proteins in cancer cells of various origins may determine the opposite AMPK/autophagy-dependent effects of SCD1 inhibition. The above observations raise the issue of the dual role of autophagy in the development of cancer. Many key autophagy-related proteins, such as Beclin 1, UV radiation resistance-associated gene (UVRAG), Bax interacting factor-1 (Bif-1), and autophagy-related (ATG), act as tumor suppressors, as their depletion leads to the development of cancer [134,135,136]. On the other hand, autophagic events promote the growth and survival of cancer cells exposed to stressful conditions (for a review, see [137]). Autophagy is also associated with the development of advanced malignancy, as the involvement of this process in cancer metastasis, chemotherapy, and in the maintenance of cancer cell stemness has been extensively studied [138,139,140,141,142,143]. Some autophagy modulators, such as rapamycin, hydroxychloroquine, and everolimus, are used in anticancer treatment [137]. Autophagy has also been shown to be tightly regulated by SCD1. SCD1 activity is required at the initial step of autophagosome formation during starvation-induced autophagy [144], for the palmitate-derived autophagy in pancreatic β-cells [145], and the autophagosome formation in *Saccharomyces cerevisiae* [146]. Since SCD1 activity is crucial for the regulation of autophagy, and at the same time autophagy is one of the mechanisms of cancer cell resistance to SCD1 inhibitors (see Section 7.2), combined therapy targeting both tumorigenic pathways should be considered. 

Since the two isoforms of SCD are expressed in human tissues (see Section 2 for details), the effects of SCD1 inhibition may appear to be compensated, to some extent, by the alternative expression of SCD5. Four isoforms of SCD (SCD1, SCD2, SCD3, and SCD4) have been identified in mice [147]. It was presented that genetic ablation of SCD1 in mice overexpressing AKT or/and neuroblastoma ras oncogene (NRas) did not inhibit lipogenesis and liver steatosis, nor did it affect the development of liver tumor, due to the upregulation of SCD2 [148]. Similarly, Rodriguez-Cuenca et al. demonstrated an alternative overexpression of the SCD3 isoform in mouse SCD1^−/−^ 3T3-L1 adipocytes, which could explain the observed maintenance of some levels of 16:1 in the membrane domains of these cells [149]. However, the authors refer to another study showing a significant decrease of the 16:1/16:0 ratio in total PLs of human SCD1 deficient adipocytes, suggesting no compensatory effect of SCD5 activity [149,150]. Furthermore, as mentioned in Section 6, the co-culture of MCF-7 and MDA-MB-231 breast cancer cells with CAFs induced the expression of SCD1 in both cancer cell lines, and the overproduction of SCD5 isoform in MCF-7 cells. Whereas pharmacological or siRNA-based inhibition of SCD1 decreased the motility of both cell lines, depletion of SCD5 did not affect the migratory ability of MDA-MB-231 cells. Interestingly, it caused a massive detachment of cultured MCF-7 cells, suggesting the protective role of SCD5 against necrotic cell death, independently of SCD1. Additionally, simultaneous silencing of SCD1 and SCD5 in MCF-7 and MDA-MB-231 did not worsen the effects observed for a single silencing of both isoforms [84,85]. Thus, in contrast to the observation in mouse cells, it is unlikely that inhibition of SCD1 would activate the alternative pathway by upregulation of SCD5 in human cancer cells, at least in the breast cancer model.

Finally, one of the latest studies presents evidence of an alternative FA desaturation pathway in cancer cells [151]. The authors observed a diversified sensitivity of cancer cells of a different origin (liver, lung, prostate, and breast) to the inhibition of SCD with Merck Frosst compound 3j. They distinguished: (1) SCD-dependent, (2) partially SCD-independent, and (3) SCD-independent cancer cells. It was shown that, in contrast to SCD-dependent cancer cells, the level of the unusual MUFA sapienate (cis-6-C16:1) was elevated in partially SCD-dependent and SCD-independent cancer cells, and increased upon administration of the SCD inhibitor. SCD inhibition also induced an increase in the level of sapienate in SCD-independent liver carcinoma (HUH7) xenografts in mice, with no effect on tumor weight. The expression level of fatty acid desaturase 2 (FADS2), an enzyme converting palmitate to sapienate, correlated with the independence of cancer cells from SCD. The sapienate/palmitate ratio indicating FADS2 activity was also significantly increased in human liver and lung cancer tissue, compared to the ratio found in blood plasma or normal tissue. Thus, the existence of an alternative desaturation pathway provides cancer cells with an increased metabolic plasticity, especially upon SCD inhibition [151]. This breakthrough discovery sheds new light on the idea of targeting desaturation pathways in cancer cells, and suggests that only combined inhibition of SCD and FADS2 can become a fully effective way of treating certain types of cancer.

## 8. SCD1 and Cancer Stem Cells

A growing body of evidence indicates that the resistance of cancer cells to chemotherapy and irradiation is determined by the presence of a population of cancer cells that possess a stem cell-like phenotype, so-called cancer stem cells (CSCs). These cells exhibit a self-renewal capability and express well-known markers of stemness, such as octamer-binding transcription factor 4 (OCT4), NANOG homeobox (NANOG), SRY-box 2 (SOX2), cluster of differentiation 24 (CD24), CD44 antigen (CD44), and prominin-1 (CD133) [87,152]. A common feature of these cells is their ability to form three-dimensional in vitro structures, called spheroids, which are useful as a suitable system for evaluating the characteristics of CSC-related solid tumors [153]. As stem cells, CSCs highly overexpress ATP-binding cassette (ABC) transporters that mediate the efflux of substances across the plasma membrane against a concentration gradient. This feature of CSCs is considered to be responsible for the resistance of tumors to anticancer agents [154]. Fulfilling the stem cell program has also been shown to contribute to the initiation of cancer cell metastasis [155]. 

Cancer stem cells were shown to play a critical role in CRC recurrence and metastasis [156]. A single-cell metabolomics study revealed significant metabolic differences between CSCs and non-CSCs that were derived from the HCT116 colorectal cancer cell line. Together with higher levels of citric acid cycle (TCA) metabolites, the higher abundance of unsaturated lipids and increase in the MUFA/SFA ratio have been observed in CSCs compared with non-CSCs. Based on these observations, a correlation analysis showed a positive relationship between the TCA metabolite, pyruvate, and C16:0, C16:1, C18:0, and C18:1 FAs. Pyruvate is converted to acetyl-CoA, the substrate of FA synthesis. Therefore, higher levels of pyruvate are expected to result in the higher production of FAs, including unsaturated FAs [152]. An analysis of FA desaturases demonstrated a dominant role of SCD1 in the synthesis of unsaturated FAs in ovarian CSC. These authors also showed that SCD1 inhibition with CAY10566 reduced the rate of proliferation of ovarian CSCs in spheroids, diminished stemness (determined by SOX2 expression), and suppressed tumor formation in a xenograft mouse model [19]. The same inhibitor suppressed the growth of glioma stem-like xenograft in mice, and significantly prolonged survival in the experimental group [90]. Furthermore, SCD1 has been shown to regulate the properties of hepatocellular carcinoma stem cells, determining their metastatic potential and resistance to sorafenib (i.e., a multikinase inhibitor that is approved for the treatment of patients with advanced HCC) [73,157]. The inhibition of SCD1 with A939572 impaired HCC stem cell self-renewal, invasiveness, and sorafenib resistance. Another orally available SCD1 inhibitor, SSI-4, which was developed at the Mayo Clinic to target HCC in vivo [92], was administered in mice that were injected with HCC cells that were derived from patients. SSI-4 reduced the growth of tumors and enhanced sorafenib toxicity with combined treatment [73] (Table 1).

A positive feedback loop was found between NF-κB, ALDH1A1, and SCD1 with regard to regulating the CSC phenotype [19,152,158]. The NF-κB pathway is a well-known pro-tumorigenic driver that is involved in maintaining cancer cell stemness. De novo-synthesized unsaturated FAs promoted the activation of NF-κB (nuclear factor κ-light-chain-enhancer of activated B cells) signaling, inducing the expression of lipid desaturases at the transcriptional level [19]. Aldehyde dehydrogenase 1 family member A1 (ALDH1A1) is an intracellular marker of CSCs that converts retinaldehyde to retinoic acid, a regulator of lipid metabolism, in favor of unsaturated FA synthesis [19]. Pisanu et al. isolated a fraction of ALDH1A1^high^ NCI-H460 cells from a NSCLC population and found a greater potential of these cells to form three-dimensional spheroids in vitro compared with ALDH1A1^low^ cells. ALDH1A1^high^ cells exhibited an increase in the expression of SCD1 and NANOG and higher resistance to cisplatin (CDDP), the first-line treatment of patients with advanced NSCLC [88]. The chemoresistance of NSCLC to CDDP arises rapidly during therapy and is considered to be driven by cisplatin-induced enrichment of the CSC population [159,160]. The spheroid-forming efficiency of ALDH1A1^high^ cells was reduced by the SCD1 inhibitor, MF-438, more profoundly compared with ALDH1A1^low^ cells, and this effect was strongly enhanced by the synergistic action of MF-438 and CDDP. The blockade of SCD1 reversed the resistance of stem-like ALDH1A1^high^ cells to cisplatin through the activation of ER stress and apoptosis. This study also showed that high levels of SCD1 were associated with an increase in the expression of CSC markers in cancer tissue that was derived from NSCLC (adenocarcinoma subtype) patients who had a more aggressive disease [88].

The Hippo pathway and its effectors, Yes-associated protein (YAP) and tafazzin (TAZ), were shown to regulate embryonic and somatic stem cell proliferation, self-renewal, and differentiation [161]. These factors are also important for the maintenance of CSCs in breast cancer and hepatocarcinoma [162,163,164]. Noto et al. found that YAP/TAZ maintains the stemness of non-small cell lung CSCs in an SCD1-dependent manner via the wingless-type MMTV integration site family (Wnt)/β-catenin pathway. The upregulation of SCD1 in spheroids resulted in the accumulation of unsaturated FAs, substrates of the acylation reaction by Porcupine (Porcn) that leads to the release of active acylated Wnt ligands from the cell and their association with cell surface receptors [165,166]. This, in turn, resulted in the stabilization of β-catenin and YAP/TAZ protein levels and their translocation to the nucleus, resulting in the activation of specific target genes that are related to stemness. The pharmacological inhibition of SCD1 with MF-438 or its depletion with siRNA increased the content of SFAs in the cell, leading to the degradation of YAP/TAZ and impairment in the formation of spheroids. Clinical data confirmed that CSCs strongly contribute to the progression of lung adenocarcinoma, in which high levels of SCD1, YAP, TAZ, and β-catenin in tumor tissue were correlated with poor patient prognosis [165]. Cross-talk between SCD1 and the Hippo pathway has also been shown to maintain the stem features and chemoresistance of melanoma CSCs. SCD1 expression was also predictive of the response of BRAF-mutated melanoma cells to BRAF and mitogen-activated protein kinase kinase (MEK) inhibitors, which are used in combination therapy for the treatment of BRAF-mutated melanoma patients [87,167,168,169,170]. The treatment of melanoma cells with these agents increased the protein levels of YAP/TAZ, coupled with the activation of YAP/TAZ target genes, such as connective tissue growth factor (*CTGF*), baculoviral IAP repeat containing 5 (*BIRC5*), cysteine rich angiogenic Inducer 61 (*CYR61*), and TEA domain transcription factor 4 (*TEAD4*). These findings indicate that SCD1 and YAP/TAZ are determinants of the resistance of melanoma cells to BRAF/MEK inhibitors. Consequently, application of the SCD1 inhibitor, MF-438, abolished the resistance of melanoma spheroids to these drugs, with simultaneous decreases in the expression of YAP/TAZ and stem cell markers [87].

Another mechanism implicating SCD1 and the Wnt/β-catenin pathway in CSC-related tumor development has been proposed by Lai et al. The authors highlighted that Wnt/β-catenin-driven expression of SCD1, and in turn, MUFA synthesis, amplifies Wnt signaling via stabilization of β-catenin and low-density lipoprotein receptor-related proteins 5 and 6 (Lrp5 and 6) mRNA in rodent hepatic stellate cells (HSCs) and tumor-initiating stem cell-like cells (TICs) from the liver [20]. Lrp family members are transmembrane receptors involved in Wnt signal transduction [171]. Genetic ablation or pharmacological inhibition of SCD1 attenuates HSC activation and self-renewal of TICs, suppressing liver fibrosis and tumor formation in mice [20].

## 9. Perspectives of SCD1 Inhibitors in Anticancer Therapy

Regardless of the wide range of SCD1 inhibitors, only few have progressed to clinical trials, almost exclusively as candidates for the treatment of type 2 diabetes [64,172]. A major obstacle to the clinical use of SCD1 inhibitors is their adverse effects, which have been broadly observed in animal models. SCD1 inactivation leads to the atrophy of sebocytes, cells that form sebaceous glands that produce sebum [173,174]. This mixture of FFAs, TAGs, wax esters, ceramides, and cholesterol is secreted onto the skin as a protective barrier against heat loss and as the eye and eyelid lubricant [175,176]. SCD1 is required for sebum production. The treatment of mice with SCD1 inhibitors caused eye dryness, hair loss, and skin dryness, leading to cold-induced hypothermia [64,174]. With regard to the use of SCD1 inhibitors as anticancer agents, many studies have reported side effects in mouse models, including mucosal discharge from the eyes, intensified squinting, and skin dysfunction after the oral administration of MF-438 [27] and A939572 [71] (Table 1), and intravenous administration of SAR707 [177]. Nevertheless, still unclear is the clinical severity of the side effects of SCD1 inhibitors. The vast majority of commonly used chemotherapeutic agents cause a wide spectrum of severe adverse symptoms, including gastrointestinal disorders, neuropathy, depression, leukopenia, and heart failure [178,179,180]. Thus, the potential benefits of the anti-cancer properties of SCD1 inhibitors may outweigh their risk of side effects, which may not necessarily be more severe than the consequences of ineffective cancer treatment. Xenon Pharmaceuticals proposed a novel triazolone derivative, pyrazolyltriazolone compound 17a. This SCD1 inhibitor had an improved adverse effect profile in rats in studies that evaluated its inhibitory potency and metabolic stability [181]. Similarly, the SCD1 inhibitor, T-3764518, had a promising in vivo response in pharmacodynamic testing and reduced the tumor growth of renal cancer xenografts in mice, without severe toxicity [83]. Finally, Ready and Nijhawan developed sebocyte-sparing SCD1 inhibitors that may be applicable in the form of a prodrug. The screening of a library of 200,000 small molecules, followed by further optimization of the screen, resulted in the selection of two molecules, the oxalamide SW208108 and benzothiazole SW203668. Both compounds had selective toxicity against the same lung cancer cell lines, but only SW203668 appeared to be bioavailable in in vivo studies and suppressed tumor growth in lung cancer xenografts in mice. SW208108 became the target of further research with regard to its selective toxicity. However, SW208108 was shown to not bind SCD1 in cell lines that were resistant to its application, indicating the notable influence of the cellular context on its activity. Further analysis showed that SW208108 is a prodrug that is irreversibly demethylated by cytochrome P450 family 4 subfamily F member 11 (CYP4F11) to dMe-SW208108. This reaction revealed a phenol group that forms covalent adducts with SCD1 [94,95]. Cytochromes of the P450 family (CYP) are frequently expressed in lung epithelial and lung cancer cells to neutralize toxic compounds [182,183]. Consistent with the above findings, the abundant expression of CYP4F11 was observed in lung cancer cell lines that were sensitive to SW208108. Furthermore, the activity of SCD1 in microsomes that were derived from the preputial gland (i.e., a specialized sebaceous gland) was unaffected by SW208108. This suggests minimal or no CYP4F11 expression in these structures. This may also explain the lack of harm of the bioavailable benzothiazole SW203668 to sebaceous glands in the mouse model. SW203668 is also metabolized by CYP4F11 to the form of an active inhibitor. Other CYP4 family members, such as CYP4F12, CYP4F22, and CYP4V2, possessed the same catalytic activity toward the examined prodrugs. An analysis of data from TCGA revealed that all four CYP4 enzymes are expressed at high levels in cancer tissue of different origins compared with corresponding healthy tissue [94]. Thus, these recently discovered CYP4 substrates may represent a novel class of cancer-targeted SCD1 inhibitors and provide a promising alternative to previously studied compounds. The repeatedly observed characteristic adverse effects of SCD1 inhibitors have been shown to be overcome by the use of metabolically activated prodrugs. Moreover, considering that most CYP enzymes are expressed in the liver and that oxalamides efficiently inhibit SCD1 in liver microsomes, the authors of this study further suggested the application of the proposed compounds to the treatment of liver-specific diseases [94]. Furthermore, Merck proposed a liver-specific SCD1 inhibitor, MK-8245, as a potential treatment of diabetes and dyslipidemia. Phase II clinical trials found no liver toxicity in the treated participants [172,184].

## 10. Conclusions

**Figure 1:**
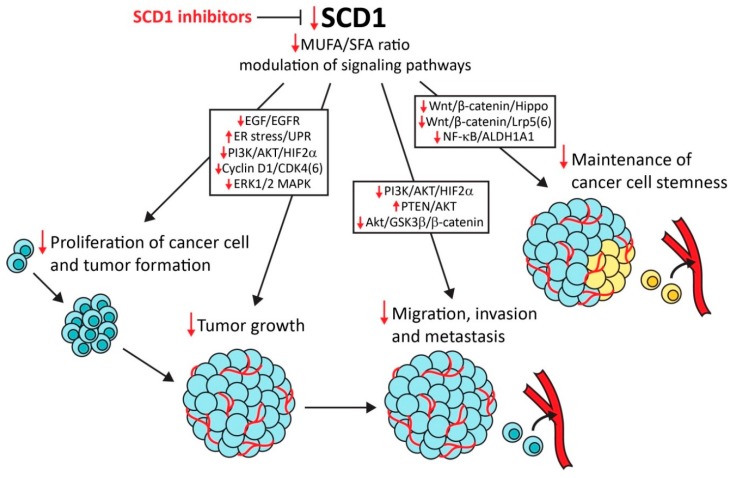
Stearoyl-CoA desaturase 1 (SCD1) as a molecular target for anticancer therapy. SCD1 inhibitors suppress cancer cell proliferation, tumor formation and growth, and cancer cell migration, invasion, metastasis, and stemness. SCD1 regulates these tumorigenic events indirectly through monounsaturated fatty acids (MUFA) synthesis or directly by modulating various signaling pathways (e.g., epidermal growth factor/epidermal growth factor receptor (EGF/EGFR) mitogenic pathway, AKT/GSKβ/β-catenin pathway for stimulation of the epithelial–mesenchymal transition, and Wnt/β-catenin/Hippo pathway for the maintenance of cancer cell stemness). Red arrows indicate the anticancer effect of SCD1 inhibition.

With regard to its crucial role in lipid metabolism, SCD1 has emerged as a main driver of abnormalities that lead to the development of metabolic disorders, such as diabetes, hyperlipidemia, hepatic steatosis, and obesity-related heart diseases [185,186]. Strong evidence indicates that SCD1 is an important determinant of cancer development and progression. In addition to its contribution to increases in cancer cell proliferation and tumor growth, SCD1 drives the development of aggressive and metastatic malignancies. Moreover, in many cancers of various origins, SCD1 is a prognostic factor for cancer progression and patient survival. Thus, SCD1 may be a promising target for anticancer therapy (Figure 1). This possibility is supported by several studies that reported the selectivity of SCD1 inhibitors to cancer cells, without affecting normal tissue. Remarkable roles of SCD1 in the maintenance of CSC stemness and promotion of cancer progression and chemoresistance have been demonstrated. These findings provide a basis for the experimental implementation of SCD1 inhibitors in combined anticancer therapy. However, most of the studied SCD1 inhibitors have not gone beyond preclinical testing because of their adverse effects in animal models. SCD1 activity is crucial for the production of sebum, and several abnormalities that result from dysfunction of the sebaceous glands have been observed in mice that are treated with SCD1 inhibitors. However, the recent discovery of metabolically activated SCD1 inhibitors [94,95] sheds new light on the possibility of the clinical use of these compounds.

## Figures and Tables

**Table 1:** Stearoyl-CoA desaturase 1 inhibitors with anticancer activity.

Inhibitor Name	Activity in Vitro	Activity in Vivo	Side Effects	References
A939572	Inhibits the proliferation and induces the apoptosis of lung, pharynx, renal, bladder, liver, colorectal, thyroid, and endometrial cancer cells. Suppresses the migration and invasion of hepatocellular carcinoma cells and breast cancer cells. Impairs the migration of breast cancer cells that is driven by cancer-associated fibroblasts. Effectively inhibits the proliferation and colony formation of clear cell renal cell carcinoma cells in combination with temsirolimus. Sensitizes glioblastoma cells to temozolomide treatment. Combined administration with bortezomib or carfilzomib impairs the proliferation and induces the apoptosis of anaplastic thyroid carcinoma cells. Impairs the self-renewal, invasiveness, and sorafenib resistance of hepatocellular carcinoma stem cells.	Suppresses the growth of gastric and colorectal cancer xenografts in mice. Combined treatment with temsirolimus effectively inhibits the growth of clear cell renal cell carcinoma xenografts in mice.	Mucosal discharge from eyes and increase in squinting. Hair loss, eye ptosis, and sebaceous gland atrophy.	[27,60,64,71,72,73,74,75,84,85,86]
MF-438	Inhibits the proliferation and induces the apoptosis of breast cancer cells. Combined administration with bortezomib or carfilzomib impairs the proliferation and induces the apoptosis of anaplastic thyroid carcinoma cells. Reverses the resistance of stem-like non-small cell lung cancer cells to cisplatin and melanoma spheroids to B-Raf proto-oncogene, serine/threonine kinase/ mitogen-activated protein kinase kinase (BRAF/MEK) inhibitors.	Combined treatment with carfilzomib suppresses the growth of anaplastic thyroid carcinoma xenografts in mice.	Mucosal discharge from eyes and increase in squinting.	[27,77,87,88]
CVT-11127	Inhibits the proliferation and induces the apoptosis of non-small cell lung cancer cells and breast cancer cells. Potentiates the gefitinib-dependent inhibition of non-small cell lung cancer cell proliferation.			[76,78,79]
CVT-12012	Potentiates the gefitinib-dependent inhibition of non-small cell lung cancer cell proliferation.			[79]
CAY10566	Inhibits the proliferation and induces the apoptosis of colorectal cancer cells. Induces the apoptosis of hepatocellular carcinoma cells. Inhibits the proliferation of ovarian cancer stem cells grown in spheroids. Impairs the stemness of ovarian cancer cells.	Suppresses lung metastasis and prolongs the overall survival of mice that are injected with co-cultured murine melanoma cancer cells and murine lung fibroblasts. Suppresses the formation of tumors by ovarian cancer stem cells in mice. Suppresses the growth of glioma stem-like xenograft in mice.		[19,29,74,89,90]
T-3764518	Inhibits the proliferation and induces the apoptosis of colorectal cancer cells.	Suppresses the growth of colorectal, mesothelioma, and renal cell adenocarcinoma xenografts in mice.	Reduces toxicity in a mouse model.	[82,83,91]
BZ36	Inhibits the proliferation of prostate cancer cells.	Suppresses the growth of gastric and prostate cancer xenografts in mice.		[81]
SSI-4	Impairs the proliferation of a broad range of cancer cells.	Reduces the growth of hepatocellular carcinoma xenografts in mice and enhances sorafenib toxicity with combined treatment. Suppresses the growth of clear cell renal cell carcinoma xenografts in mice.		[73,92,93]
SW208108	Induces the apoptosis of non-small cell lung cancer cells.		Pro-drug does not affect sebocytes.	[94,95]
SW203668	Induces the apoptosis of non-small cell lung cancer cells.	Suppresses the growth of non-small cell lung cancer xenografts in mice.	Pro-drug does not affect sebocytes.	[94,95]
